# Supplementary material for: SIK2 Controls the Homeostatic Character of the POMC Secretome Acutely in Response to Pharmacological ER Stress Induction
Source: Cells. 2024 Sep 17;13(18):1565. doi: 10.3390/cells13181565 (PMC11430698; doi:10.3390/cells13181565)
Supplement: Supplementary file 1 [file cells-13-01565-s001.zip › cells-3166537-supplementary.pdf]

Supplementary Material

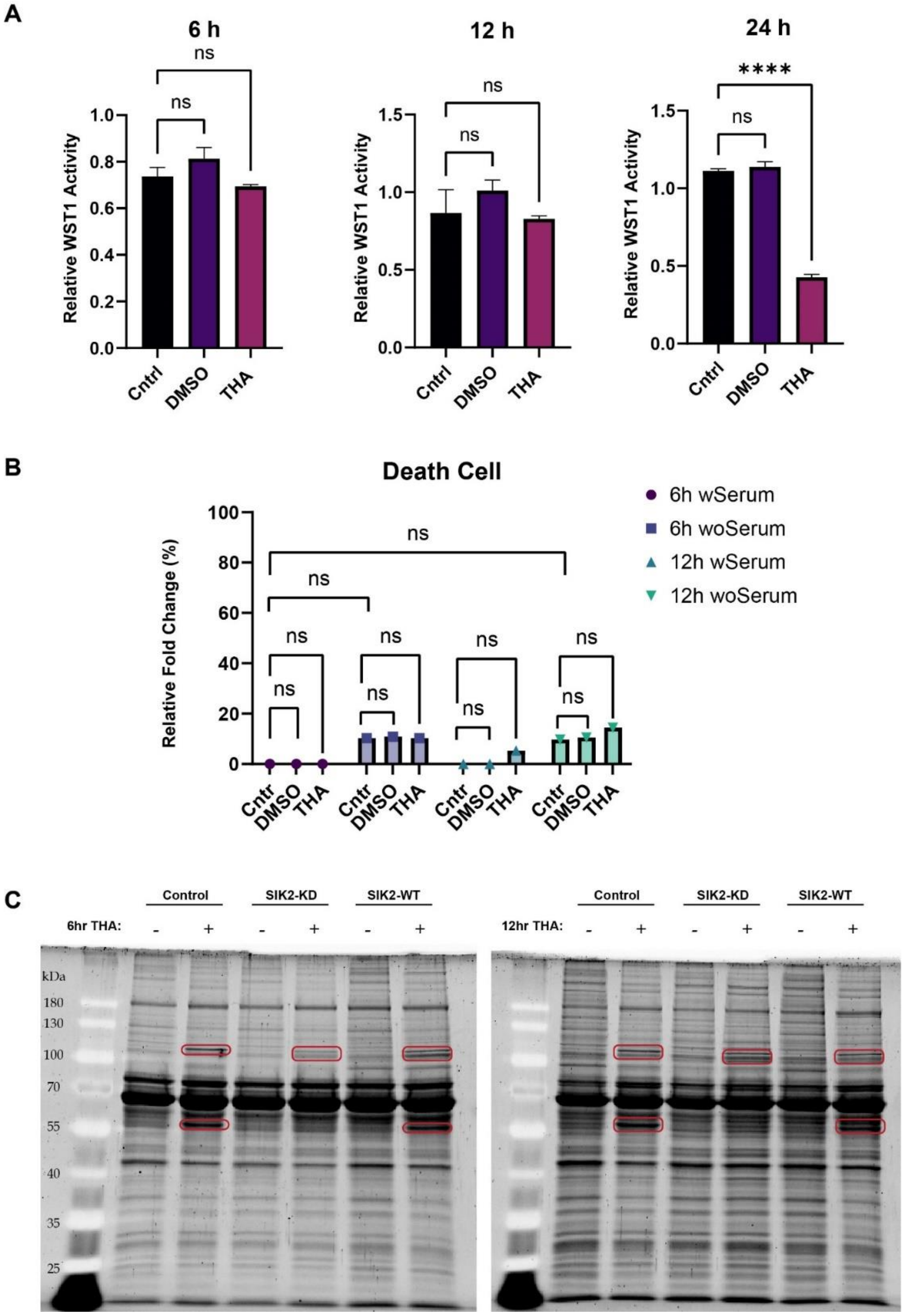

Supplementary Figure S1. Evaluation of cytotoxicity and cell viability against stress conditions, and quality control of POMC secretomes. **A)** To investigate cell proliferation, WST-1 proliferation assay was performed, all conditions were compared to control (*n*=9). **B)** The impact of serum deprivation on cellular survival was evaluated through conventional viable cell counting with trypan blue staining for up to 12 hours. All experimental conditions were normalized to the control group of 6 hours with serum (*n*=9). **C)** Purified secretomes from control, SIK2 knockdown (SIK2 KD), and SIK2 overexpressing (SIK2 WT) POMCs under the ER stress (6 h and 12 h), were subjected to 1D electrophoresis and analyzed after total protein staining with SybroRuby gel stain. The red rectangular indicator denotes bands of proteins that are differentially secreted. Error bars are means ± standard deviation (SD), ****$P < 0.0001$, ns, non-significant.

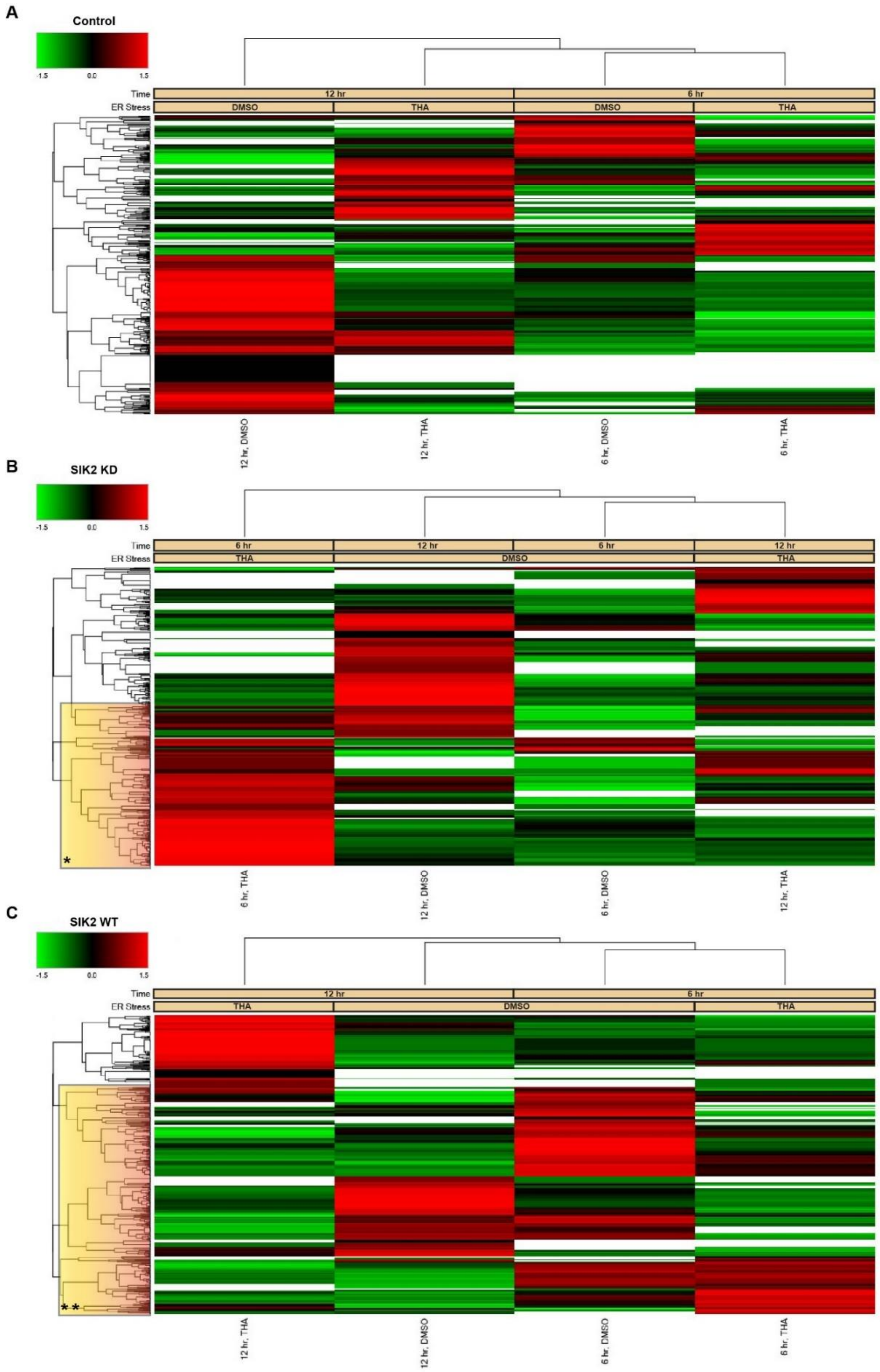

Supplementary Figure S2. Heat maps for ER stress responses specific to SIK2 manipulations. Heat map of POMCs secretome proteins across ER stress conditions (6 hr and 12 hr) to scrambled **(A)**, SIK2 KD **(B)**, and SIK2 WT **(C)** from hierarchical clustering using Proteome Discoverer 2.3. Rows represent proteins and columns represent groups of replicates. Colors correspond to z-scores (green shows the decrease; black shows no change; red shows the increase). * Proteins with the highest upregulation at 6 hours THA in the SIK2 KD POMCs secretome. ** Proteins with the highest upregulation at 6 hours DMSO in the SIK2 WT POMCs secretome.

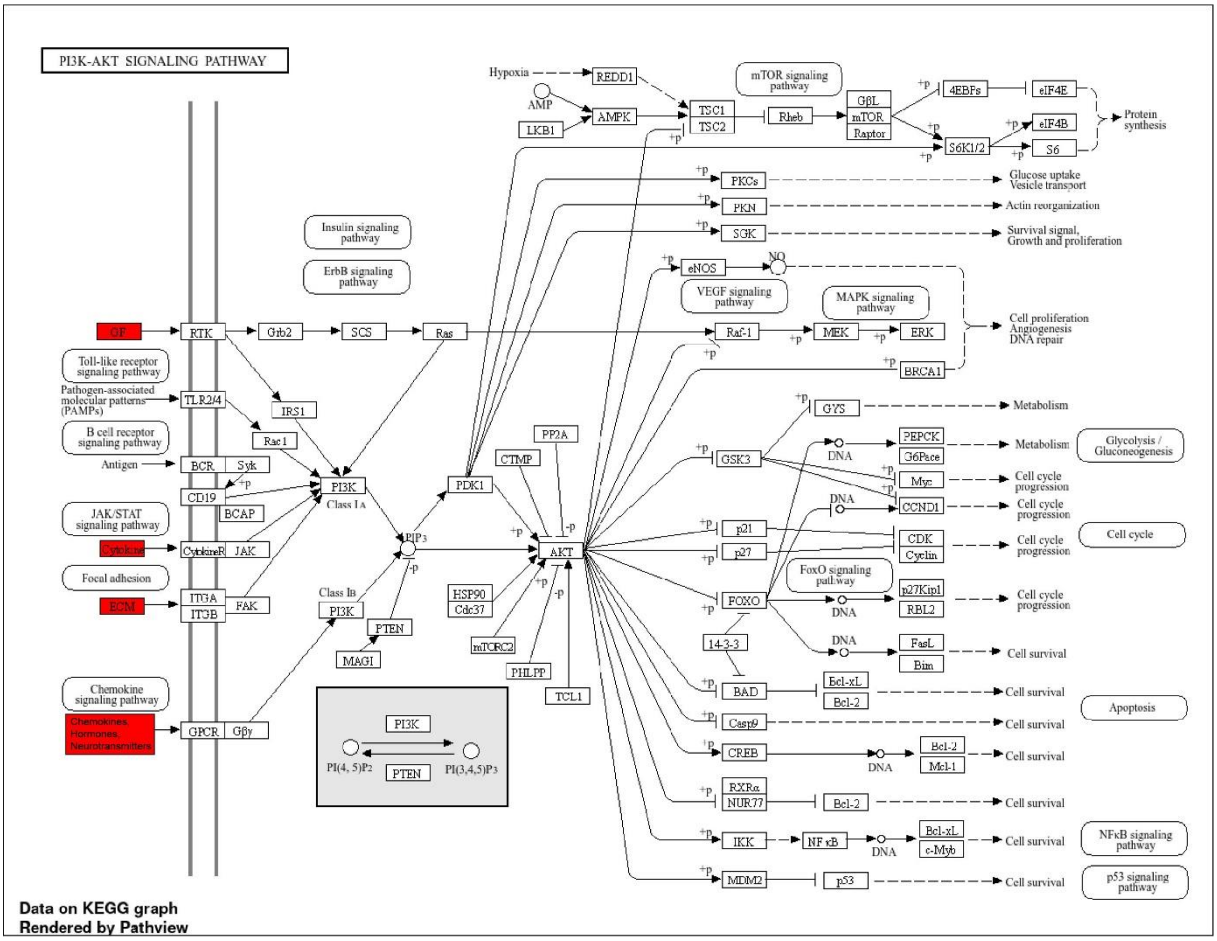

Supplementary Figure S3. PI3K-AKT pathway with detected proteins according to the KEGG database. The PI3K-AKT pathway belongs to both Cluster 2 and Cluster 4 of the functional enrichment analysis of differentially secreted proteins. The illustration is generated by ShinyGo and shows related terms in red rectangles.

Supplementary Table S1: SIK2 and prolonged ER stress-dependent Relative Fold Changes of three major clusters

					Relative Abundance Ratio						
					12hr ER stress response (THA/DMSO) [a]			12hr Stress independent (DMSO)		12hr Stress dependent (THA)	
	Accession ID	Protein Name (Abbreviation)	FDR Class*	HeatMap Cluster	Cntrl	KD	WT	KD/Cntrl [b]	WT/Cntrl [c]	KD/Cntrl [b]	WT/Cntrl [c]
Obesity and Feeding Behavior	O35082	Klotho (**Kl**)	Low	C1-C5	0,315	1,211	1,066	1,12	1,225	4,396	4,15
	Q543J5	Antithrombin-III (**SerpinC1**)	Medium	C1-C5	1,062	0,785	1,184	1,174	0,896	0,886	1,072
	Q9Z0J7	Growth/Differentiation Factor 15(**Gdf15**)	Low	C2-C5	0,348	0,953	0,957	1,179	0,702	1,303	1,274
	Q62361	Pro-thyrotropin-releasing hormone (**Trh**)	Low	C2-C5	100	12,618	10,397	0,886	1,005	1,239	0,961
	B7ZMY9	Erythropoietin (**Epo**)	Low	C2-C4	1	1	30,091	10,696	1	0,716	14,771
	B2MWM9	Calreticulin (**Calr**)	High	C1-C5	7,743	2,196	26,093	0,254	0,527	0,089	2,485
	P12023	Amyloid-beta precursor protein (**App**)	Medium	C2-C6	0,546	0,939	0,799	1,032	0,957	1,073	1,243
	P15379	CD44 antigen (**Cd44**)	High	C1-C4	1,499	1,752	1,419	1,945	1,003	2,169	0,607
	Q71LX8	Heat shock protein HSP 90-beta (**Hsp90ab1**)	High	C3-C5	0,378	1,076	1,265	0,545	0,87	0,72	1,319
	Q9Z1P8	Angiopoietin-related protein 4 (**Angptl4**)	Low	C2-C5	0,466	0,609	1,783	1,435	1,355	1,594	2,578
	P12032	Metalloproteinase inhibitor 1(**Timp1**)	High	C2-C4	0,053	0,077	0,083	0,95	0,827	1,288	1,136
	P25785	Metalloproteinase inhibitor 2 (**Timp2**)	High	C2-C5	0,119	0,178	0,29	0,938	0,812	1,424	1,673
	O35988	Syndecan-4 (**Sdc4**)	Medium	C1-C5	1,007	1,22	0,988	0,237	0,685	0,397	1,408
	Q5NCU4	SPARC (**Sparc**)	High	C2-C6	0,052	0,034	0,034	1,053	0,734	0,882	0,484
	A0A338P7H5	Alpha-2-HS-glycoprotein (**Ahsg**)	High	C3-C5	1,35	0,699	1,143	1,425	0,533	0,357	0,727
	Q640N1	Adipocyte enhancer-binding protein 1 (**Aebp1**)	High	C2-C4	0,02	0,062	0,037	0,554	1,213	1,122	1,404
Axonal Guidance & Neurite Outgrowt	O89029	Matrilin-4 (**Matn4**)	Low	C1-C6	1,135	0,695	0,841	1,593	1,048	0,508	0,828
	Q9QZF2	Glypican-1 (**Gpc1**)	Medium	C2-C6	0,409	0,01	1,832	0,069	0,389	0,052	1,706
	P70275	Semaphorin-3E (**Sema3e**)	Low	C2-C5	0,567	0,28	1	2,388	0,091	1,604	0,01
	Q9QZL9	Dickkopf-like protein1 (**Dkkl1**)	Low	C2-C4	0,994	1,899	2,188	1,061	0,763	2,106	0,993
	P10605	Cathepsin B (**Ctsb**)	High	C3-C6	0,115	0,158	0,095	0,95	0,892	1,339	0,571
	A0A0G2JE75	Bone morphogenetic protein 3 (**Bmp3**)	Low	C3-C5	0,702	10,993	1,037	0,11	0,823	1,046	1,33
	P20722	Bone morphogenetic protein 6 (**Bmp6**)	Low	C1-C6	12,397	0,59	29,513	1,556	0,488	0,077	1,858
	P11087	Collagen alpha-1(I) chain(**Col1a1**)	High	C2-C4	0,187	0,264	0,246	1,295	0,796	1,857	0,678
	Q01149	Collagen alpha-2(I) chain (**Col1a2**)	High	C2-C5	0,116	0,445	0,148	1,317	1,112	4,769	1,005
	Q04857	Collagen alpha-1(VI) chain (**Col6a1**)	High	C2-C6	0,321	0,719	0,543	0,649	0,642	1,229	0,989
	B1B0C7	Perlecan (heparan sulfate proteoglycan 2)(**Hspg2**)	High	C1-C6	0,156	0,15	0,254	0,717	0,736	0,607	1,133
	P10493	Nidogen-1 (**Nid1**)	High	C2-C4	0,09	0,081	0,256	0,787	0,751	0,782	1,981
Inflammation	Q58EV5	High mobility group protein B1 (**Hmgb1**)	Medium	C2-C5	0,772	1,169	0,823	1,103	1,067	1,204	1,131
	P34884	Macrophage migration inhibitory factor(**Mif**)	High	C2-C4	1,428	1,822	1,709	1,382	0,896	2,248	1,073
	O35188	Fractalkine (**Cx3cl1**)	Medium	C1-C6	0,931	0,969	1,353	1,257	1,13	0,642	0,92
	Q8R460	Interleukin-36 gamma (**Il36g**)	Medium	C1-C6	1,016	0,759	0,882	1,359	0,972	0,676	0,844
	Q9CPT4	Myeloid-derived growth factor(**Mydgf**)	High	C2-C4	15,375	6,446	23,569	1,148	0,581	0,486	0,457
		Annexin (**Anxa1**)	Medium	C2-C5	1,001	1,086	1,344	0,53	0,548	0,888	0,585
		Granulin (**Grn**)	High	C2-C6	0,176	0,265	0,216	0,859	1,283	1,431	0,737

*FDR class, High: q value ≤0.01; Medium: q value ≤0.05; Low: q value >0.05.
For proteins with a fold change ≥ 2: P Adj. ≤ 0.05.

a: The abundance of secreted proteins in ER stressed cells compared to that of unstressed cells.

b: The abundance of secreted proteins in SIK2 knockdown cells compared to control cells.

c: The abundance of secreted proteins in SIK2 overexpressing cells compared to control cells.
